# Considerations on the human Achilles tendon moment arm for in vivo triceps surae muscle–tendon unit force estimates

**DOI:** 10.1038/s41598-020-76625-x

**Published:** 2020-11-11

**Authors:** Denis Holzer, Florian Kurt Paternoster, Daniel Hahn, Tobias Siebert, Wolfgang Seiberl

**Affiliations:** 1 Department of Sport and Health Sciences, Biomechanics in Sports, Technical University of Munich, Munich, Germany; 2 Human Movement Science, Ruhr University Bochum, Faculty of Sport Science, Bochum, Germany; 3 School of Human Movement and Nutrition Sciences, University of Queensland, Brisbane, Australia; 4 Department of Motion and Exercise Science, University of Stuttgart, Stuttgart, Germany; 5 Department of Human Sciences, Human Movement Science, Bundeswehr University Munich, Neubiberg, Germany

**Keywords:** Physiology, Anatomy

## Abstract

Moment arm-angle functions (MA-a-functions) are commonly used to estimate in vivo muscle forces in humans. However, different MA-a-functions might not only influence the magnitude of the estimated muscle forces but also change the shape of the muscle’s estimated force-angle relationship (F-a-r). Therefore, we investigated the influence of different literature based Achilles tendon MA-a-functions on the triceps surae muscle–tendon unit F-a-r. The individual in vivo triceps torque–angle relationship was determined in 14 participants performing maximum voluntary fixed-end plantarflexion contractions from 18.3° ± 3.2° plantarflexion to 24.2° ± 5.1° dorsiflexion on a dynamometer. The resulting F-a-r were calculated using 15 literature-based in vivo Achilles tendon MA-a-functions. MA-a-functions affected the F-a-r shape and magnitude of estimated peak active triceps muscle–tendon unit force. Depending on the MA-a-function used, the triceps was solely operating on the ascending limb (n = 2), on the ascending limb and plateau region (n = 12), or on the ascending limb, plateau region and descending limb of the F-a-r (n = 1). According to our findings, the estimated triceps muscle–tendon unit forces and the shape of the F-a-r are highly dependent on the MA-a-function used. As these functions are affected by many variables, we recommend using individual Achilles tendon MA-a-functions, ideally accounting for contraction intensity-related changes in moment arm magnitude.

## Introduction

For a better understanding of muscle mechanics, muscle function, joint loading and control of human locomotion it is crucial to reliably predict in vivo muscle forces. Direct force measurements within the muscle or along the force transmitting tendons are highly invasive and thus rarely used in human muscle research^1–6^. More typically, in vivo muscle forces are derived from external joint torques, which can be measured on strength testing machines or estimated through inverse dynamics. From these measured/estimated net joint torques, it is then possible to estimate muscle–tendon unit forces by dividing the torque by the muscle’s moment arm (MA). Therefore, the MA has a considerable influence on the magnitude of the calculated forces.

One of the most important muscle groups for plantarflexion, and therefore for locomotion^7–9^, is the triceps surae. Its forces are transmitted by the Achilles tendon (AT) to the calcaneus bone. In vivo, the AT MA is usually determined via one of three methods. (1) The geometric imaging method, where magnetic resonance imaging or radiography is used to measure the perpendicular distance between the center of rotation of the tibio-talar joint and the AT line of action. This is either done exclusively in the sagittal plane^10–13^ or in three dimensional space^10,14^. (2) The tendon excursion method, which assesses tendon displacement via ultrasound due to ankle joint rotation during passive movement. The MA is then calculated as the slope of the tendon displacement versus the ankle joint rotation^15^. (3) A geometric imaging method using motion tracking and ultrasound (UsKin). Thereby, an ultrasound probe (calibrated in space) is used to track the AT line of action^16–27^. Similar to the geometric imaging method, the AT MA is calculated as the perpendicular distance between the AT line of action and the ankle joint center of rotation.

It is well established that throughout a joint’s range of motion, the muscle specific MA is not a fixed value but rather a function of the joint angle. Various AT moment arm-angle functions (MA-a-function) have been proposed in the past, however, there is no gold standard function to use when calculating in vivo triceps surae muscle–tendon unit forces (from here TS refers to the entire triceps surae muscle–tendon unit). This could potentially be problematic for the comparison and interpretation of results as different assumptions regarding the AT MA-a-function might not only significantly influence the magnitude of calculated forces but also potentially affect the estimated shape of the force-angle relationship (F-a-r). This has recently been shown by Bakenecker et al.^28^ for the knee extensor muscles when comparing different literature based patella tendon MA-a-functions. Accordingly, we hypothesized that the shape and maximum force of the TS F-a-r would also differ between different AT MA-a-functions found in the literature.

## Methods

### Participants

Fourteen males (age 29 ± 6 years, height: 180 ± 4 cm, body mass 79 ± 5 kg), free of any musculoskeletal and neuronal impairments in the right lower limb, voluntarily participated in the present study after providing their free written informed consent. The experimental protocol was approved by the Faculty of Sport Science Ethics Committee at Ruhr University Bochum and conducted according to the Declaration of Helsinki.

### Experimental setup

Participants performed right-legged maximum voluntary fixed-end plantarflexion contractions (MVCs) on an isokinetic dynamometer (IsoMed2000, D&R Ferstl, GmbH, Hemau, GER). Participants lay in a prone position with their knee and hip fully extended. The participant’s right foot was tightly strapped to a customized foot plate, attached to the dynamometer’s lever, to avoid heel displacement. The rotational axis of the dynamometer was carefully visually aligned with the ankle’s axis of rotation at rest.

Participants attended one familiarization session to get used to the setup. Their ability to produce good quality MVCs was checked by the principal investigator. Thereby, criteria were (1) reproducibility of peak torque and (2) shape of the torque-time curve (steady plateau; checked visually). Prior to the measurement, each participant performed a standardized warm-up (ten submaximal eccentric contractions, ten submaximal concentric contractions and three MVCs) to precondition the muscle–tendon unit^29^.

### Determination of individual torque–angle relationship

In a randomized order, plantarflexion MVCs were performed with the dynamometer set to 20° and 10° plantarflexion (PF), 0° (referring to the neutral position with the sole of the foot perpendicular to the shank), and 5°, 10°, 15°, 20°, 25° and 30° dorsiflexion (DF). For each position, the peak active torque out of two trials was used for analysis.

To account for soft tissue compression and deformation of the foot plate during MVCs, ankle joint angles of the right foot were recorded by a ten-camera motion analysis system at 200 Hz (Vicon Peak, Oxford, UK) synchronized to the dynamometer (1000 Hz). Reflective markers (9 mm) on predefined anatomical landmarks defined 3 segments (foot, shank, thigh). The anatomical landmarks were: lateral malleolus, medial malleolus, lateral femoral condyle and medial femoral condyle (most medial/lateral aspect respectively), trochanter, first and fifth metatarsophalangeal joint. Data were captured using the Vicon Nexus software (Oxford Metrics, Oxford, UK) and then transferred into MATLAB (The MathWorks, Inc., Natick, Massachusetts, United States) for further analysis. Joint angles were defined relative to the segment angles measured in the standardized static reference position (knee fully extended, shank longitudinal axis perpendicular to the sole of the foot). Additional three markers were placed on the bottom of the foot plate attached to the dynamometer lever to control for material deformation and heel lift (change in distance between malleoli and foot plate) during MVC.

**Figure 1:**
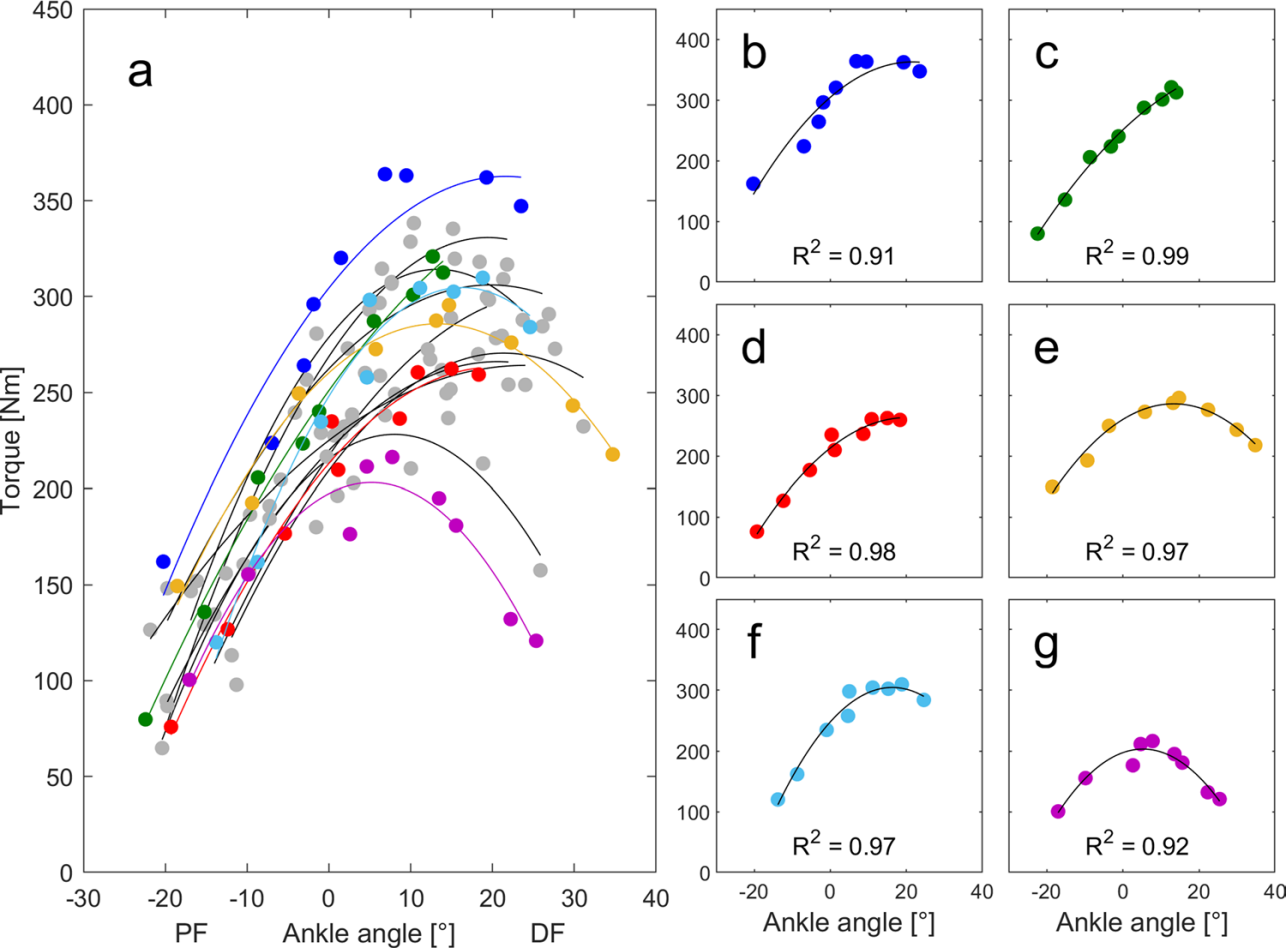
Measured and fitted torque–angle relationships. (**a**) Individual torque–angle relationships derived from maximum fixed-end plantarflexion contractions for all participants. (**b**–**g**) Subplots of six individual examples with corresponding coefficients of determination (R^2^). Note that (**e**–**g**) exhibit a pronounced descending limb of the torque–angle relationship, whereas (**b**–**d**) do not show this. (0° ankle joint angle refers to neutral foot position; *DF* dorsiflexion, *PF* plantarflexion angles).

All participant-specific torque–angle relations were fitted using a polynomial fit (2nd degree, 30). According to these fits, individual angle specific peak active torques were determined between 20° PF and 25° DF (Fig. 1).

### Calculation of TS force

TS force was calculated from the plantarflexion torque divided by the angle-specific MA and weighted according to its anatomical proportion of the plantar flexors (~ 77% based on the physiological cross-sectional area^7^). The ankle joint angle-specific AT MA-a-functions used in this study were taken from the data of 17 previously-published in vivo studies (Table 1). If AT MA values were not directly available from text or tables, but presented in graphs (indicated in Table 1), ImageJ (ImageJ v.1.48; National Institutes of Health, USA) was used to estimate the joint angle specific MAs^10^. All MA-a-functions were fitted using a polynomial fit (2nd degree)^30^ including all available data points between 30° PF and 25° DF (parameters of the polynomials are presented in Table 2). For each MA-a-function, group mean and participants’ individual F-a-r were calculated for statistical analysis.

**Table 1:** Summary of the literature based in vivo Achilles tendon moment arm functions and their method of moment arm determination used for estimation of triceps surae muscle–tendon unit force. Note: Peak force estimates are based on the experimental data of the current study.

Study	Subjects M/F = male/female	Body height (cm)	Method	Contraction intensity	Peak force (N)
Deforth et al.^35^	40M, 59F (33)^a^	–	GI (1 frame)	Rest	8083 ± 1943
Fath et al.^13^	7M, 2F	180 ± 10	GI (2D)	Rest	5130 ± 1194
Fath et al.^13^	7M, 2F	180 ± 10	TE	Rest	6327 ± 1394
Fletcher et al.^34^	6M, 2F	169 ± 11	TE	Rest	6944 ± 1436
Hashizume et al.^10^	15M	172 ± 5	GI (3D finite helical angle)	Rest	9947 ± 2194
Hashizume et al.^10^	15M	172 ± 5	GI (2D)	Rest	5165 ± 1245
Maganaris et al.^12^	6M	175 ± 8	GI (2D)	MVC	4312 ± 1026
Maganaris et al.^12^	6M	175 ± 8	GI (2D)	Rest	5347 ± 1253
Maganaris et al.^37^	6M	175 ± 8	TE	MVC	5080 ± 1234
Maganaris et al.^37^	6M	175 ± 8	TE	Rest	5003 ± 1203
Manal et al.^36^	10M	177 ± 5	UsKin	Rest	6009 ± 1245
Manal et al.^36^	10M	177 ± 5	UsKin	MVC	5735 ± 1177
Obst et al.^19^	9M, 7F	171 ± 10	UsKin (straight)	Rest	5559 ± 1297
Obst et al.^19^	9M, 7F	171 ± 10	UsKin (curved)	Rest	5591 ± 1299
Rugg et al.^11^	10M	180 ± 7	GI (2D)	Submaximal	5009 ± 1160
Clarke et al.^14^	5M, 5F		GI (3D finite helical axis)	Rest	
Sheehan et al.^33^	14M, 6F		GI (2D IHA)	Rest	
Sheehan et al.^33^	14M, 6F		GI (3D IHA)	Rest	

Experimental data from Sheehan et al.^33^ 2D and 3D, and Clark et al.^14^ are presented, but were not considered in further analysis.

*GI* geometric imaging, *TE* tendon excursion, *UsKin* combination of ultrasound and motion tracking, *IHA* instantaneaous helical angle.

^a^Only the function for the normal foot including 33 participants was considered in this study.

**Table 2:** Polynomial fits (second order) and coefficients of determination (R^2^) for the 15 literature-based Achilles tendon moment arm functions used for the calculation of the force–angle relationship.

Study	AT MA_θ_ = p1 × θ^2^ + p2 × θ + p3				Ankle angles measured
	p1	p2	p3	R^2^	(°)
Deforth et al.^35^^a^	− 0.835e − 3	− 0.0572	4.560	0.9997	function (0)
Fath et al.^13^	− 0.478e − 3	− 0.0308	5.177	0.9998	− 30, − 15, 0, 15
Fath et al.^13^	− 0.011e − 3	− 0.0049	3.529	0.9676	− 30, − 15, 0, 15
Fletcher et al.^34^^b^	− 0.773e − 3	− 0.0005	3.532	0.5251	− 20, − 15, − 10, − 5, 0, 5, 10, 15, 20, 25
Hashizume et al.^10^	− 1.750e − 3	− 0.0325	4.025	0.9495	− 20, − 10, 0, 10
Hashizume et al.^10^	− 7.143e − 19	− 0.0340	4.930	0.9966	− 20, − 10, 0, 10
Maganaris et al.^12^	− 0.222e − 3	− 0.0397	6.017	0.9972	− 30, − 15, 0, 15
Maganaris et al.^12^	− 0.222e − 3	− 0.0260	4.730	0.9966	− 30, − 15, 0, 15
Maganaris et al.^37^	0.248e − 3	− 0.0328	4.832	1.000	− 37.5, − 22.5, − 7.5, 7.5, 22.5
Maganaris et al.^37^	0.227e − 3	− 0.0294	4.833	0.9666	− 37.5, − 22.5, − 7.5, 7.5, 22.5
Manal et al.^36^	− 0.300e − 3	0.0040	3.646	0.9250	− 20, − 10, 0, 10, 20
Manal et al.^36^	− 0.179e − 3	0.0057	3.748	0.9756	− 20, − 10, 0, 10, 20
Obst et al.^19^	− 0.027e − 3	− 0.0193	4.303	0.9943	− 20, − 15, 0, 5, 10
Obst et al.^19^	− 0.029e − 3	− 0.0179	4.253	0.9949	− 20, − 15, 0, 5, 10
Rugg et al.^11^^c^	− 0.515e − 3	− 0.0294	5.263	0.9903	− 30, − 20, − 15, − 10, 0, 10

The angle specific Achilles tendon moment arm (AT MA_θ_) was calculated as a function of the ankle joint angle (θ).

^a^Only the AT MA function for a normal foot was considered in this study.

^b^Only the corrected AT MA values were considered in this study.

^c^AT MA values were taken from the table presented by Hashizume et al.^10^. Ankle joint angles: 0° refers to neutral foot position; positive and negative angles represent dorsiflexion and plantarflexion, respectively.

### Electromyography (EMG) and ultrasound measurements

Muscle activities of gastrocnemius lateralis, soleus und tibialis anterior were recorded at 1000 Hz (OT bioelettronica, Italy) through adhesive Ag/AgCl surface electrodes (H124SG, CovidienTM, Germany), placed according to the SENIAM recommendations^31^. Fascicle length of the gastrocnemius medialis (GM) was recorded via ultrasound at 61.5 Hz (Echoblaster 128, UAB Telemed, Lithuania) using a 60 mm linear probe (7 MHz; image depth 50 mm), carefully placed at ~ 50% of GM length over the midlongitudinal axis. A voltage signal generated by the ultrasound systems was used to synchronize the measurement systems.

### Data processing

Measured ankle joint torque and angular data were smoothed (4th order dual pass low-pass filter; 10 Hz) and torques were corrected for angle specific gravitational and passive torque. For this, the participants’ foot was slowly rotated (5°/s) by the dynamometer throughout the tested range of motion (three times in each direction) with the EMG system controlling for any muscular activity. Thereby, passive rotations were invalid/repeated when the EMG signal during a passive rotation of any tested muscle was greater than the EMG signal of the corresponding muscle during resting trials plus 2 standard deviations. To calculate active torque, the mean angle specific passive torque was then subtracted from the corresponding angle specific measured peak MVC torque.

EMG data was offset corrected, filtered (4th order bandpass; 10–500 Hz), full-wave rectified and smoothed (300 ms moving average). For each of the analyzed muscles, the peak smoothed EMG signal out of all individual MVCs served for EMG normalization. Tibialis anterior activity was normalized to the peak activity measured during three additional dorsiflexion MVCs at 20° DF, where tibialis anterior muscles force potential is close to its maximum^32^.

In each trial, GM fascicle length was manually measured in the video frame corresponding to the instance of peak torque. This was done by two raters (three times each) using the “Straight Line Tool” in ImageJ. Thereby, a line was aligned parallel to the GM fascicle orientation between the deeper boundary of the upper aponeurosis and the upper boundary of the deep aponeurosis. Mean fascicle length was used for statistical analysis.

### Statistical analysis

The coefficient of determination (R^2^) was used to assess the goodness of individual and mean polynomial fits of all literature-based AT MA-a-functions. One-way repeated measures analysis of variance (ANOVA) and post-hoc comparisons with Bonferroni adjustments were performed to identify significant differences in (1) estimated maximum force across different AT MA-a-functions and (2) muscle activity in the tested muscles across the tested ankle joint positions. In case sphericity assumptions were violated, the Greenhouse–Geisser correction was used. A two-way repeated measures ANOVA was used to examine differences in maximum TS forces at each ankle angle across AT MA-a-functions (within-method comparison; ankle position × MA-a-function). With a significant main effect of ankle joint angle on TS force, repeated contrasts were analyzed to test for significant differences in force between successive ankle joint configurations (i.e. 10°–15° DF, 15°–20° DF) to identify ascending, descending or plateau region on the F-a-r (p > 0.05). All statistical tests were performed using the Statistical Package for the Social Sciences (IBM SPSS, Chicago, IL, USA) with the level of significance set at α = 0.05.

## Results

**Figure 2:**
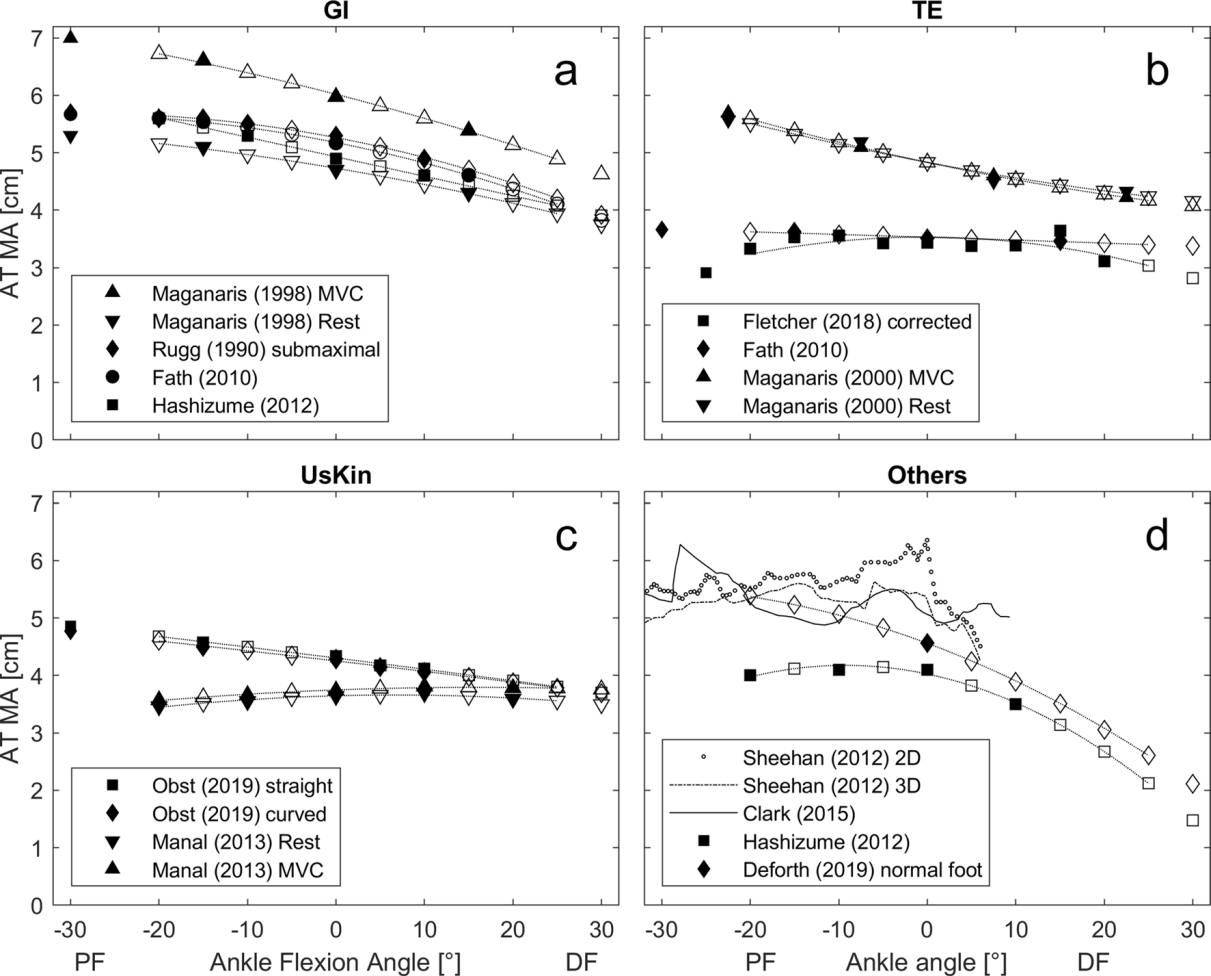
Literature-derived in vivo Achilles tendon moment arm angle functions separated by their method of moment arm determination. (a) (*GI* geometric imaging, (b) *TE* tendon excursion, (c) *UsKin* combination of ultrasound and motion tracking, for more information see Table 1. Filled symbols represent ankle joint positions measured in the associated study. Blank symbols represent the calculated AT MA by the polynomial fits (dotted line). (0° ankle joint angle refers to neutral foot position; *DF* dorsiflexion, *PF* plantarflexion angles). Please note: experimental data from Sheehan et al.^33^ 2D and 3D, and Clark et al.^14^ are presented in (**d**), but were not considered in further analysis.

All results in the figures, tables and text are presented as mean ± standard deviation. The AT MA-a-functions by Clark et al.^14^ and Sheehan et al.^33^ were discarded from further analysis, as the high variability in the data in addition to the rather small tested range of motion (Fig. 2d) did not allow the determination of a suitable fit that would reliably predict AT MAs throughout the tested range of motion. For the AT MA-a-function provided by Fletcher et al.^34^ coefficient of determination was rather low (R^2^ = 0.53). Mean R^2^ for the polynomial fits of the remaining 14 AT MA-a-functions was 0.99 ± 0.02 with R^2^ ranging between 0.93 and 1.00 (Table 2).

We found displacements of the dynamometer’s lever of up to 4.1° (on average 1.9 ± 0.8°) during MVCs. Further, the inevitable heel lift during MVCs was on average 1.3 ± 2.1 mm. This partly explains the differences between kinematics-based ankle angles ranging from 18.3 ± 3.2° PF to 24.2 ± 5.1° DF, compared to the dynamometer defined range of motion (20° PF to 30° DF).

**Figure 3:**
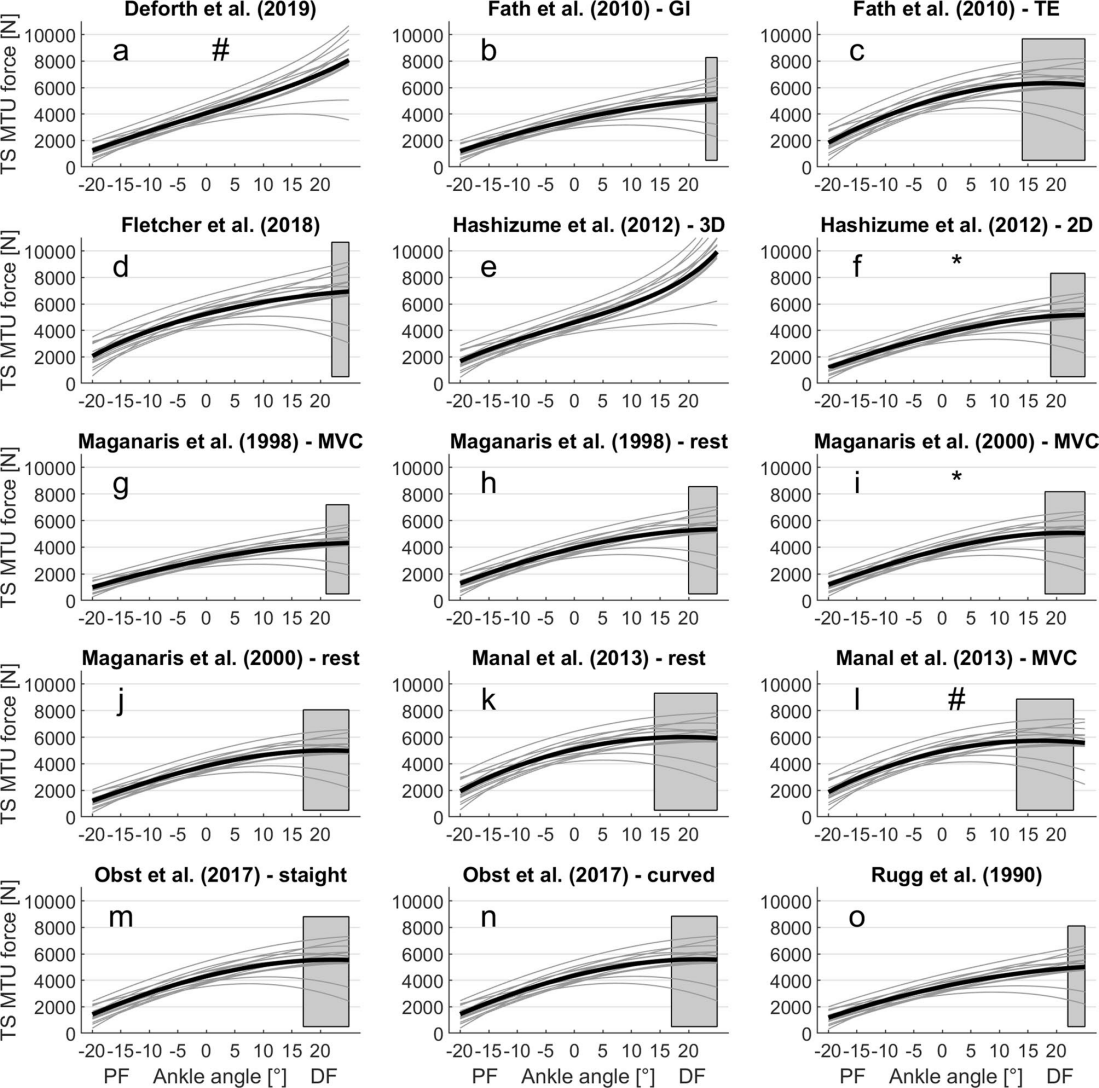
Estimated force-angle relationships (F-a-r) of the triceps surae muscle–tendon unit. Participant specific (grey lines) and mean (black line) F-a-r estimated from various literature-based in vivo Achilles tendon moment arm angle functions (AT MA-a-functions). The grey shaded area marks the plateau region of the mean F-a-r with no differences in neighboring force level (p > 0.05). Ankle angles left/right of the plateau region represent the ascending/descending limbs of the F-a-r (p < 0.05), respectively. Subplot titles indicate the AT MA-a-function used for the estimation of triceps muscle–tendon unit forces (see also Table 1). Identical symbols (*, #) indicate which MA-a-functions do not lead to statistically significant differences in calculated force. (0° ankle joint angle refers to neutral foot position; *DF* dorsiflexion, *PF* plantar flexion angles).

Individual torque–angle relationships and respective fits are presented in Fig. 1. Mean R^2^ of the fits was 0.94 ± 0.04, ranging between 0.86 and 0.99 (Fig. 1). Maximum TS forces across AT MA-a-functions were significantly different (main factor: p < 0.001) ranging from 4312 ± 1156 to 9947 ± 2194 N. The two-way ANOVA revealed significant main effects of ankle position (p < 0.001), AT MA-a-function (p < 0.001), as well as an interaction (p < 0.001) on estimated forces. Post hoc tests revealed no differences between TS force estimation for the MA-a-function by Deforth et al.^35^ and Manal et al.^36^ and also no difference between TS force estimation for the MA-a-function by Maganaris et al.^37^ and Hashizume et al.^10^ (Fig. 3).

The F-a-r as calculated by the included AT MA-a-functions are presented in Fig. 3. Repeated contrasts revealed that the AT MA-a-functions proposed by Deforth et al.^35^ and Hashizume et al.^10^ showed the TS to be working solely on the ascending limb of the F-a-r (Fig. 3a,e). The remaining 13 AT MA-a-functions all resulted in a TS force plateau (i.e. no significant difference between the TS forces at adjacent ankle joint angles) towards the more dorsiflexed positions starting between 13° and 23° DF. Only the AT MA-a-function fitted on the data provided by Manal et al.^36^ resulted in a small descending limb of the TS force-angle relationship starting at 23° DF (Fig. 3l). Muscle activity of the tibialis anterior and gastrocnemius lateralis did not show significant differences at different ankle joint positions. The ANOVA revealed a significantly reduced EMG signal of the soleus (p < 0.05) at the most plantarflexed position.

**Figure 4:**
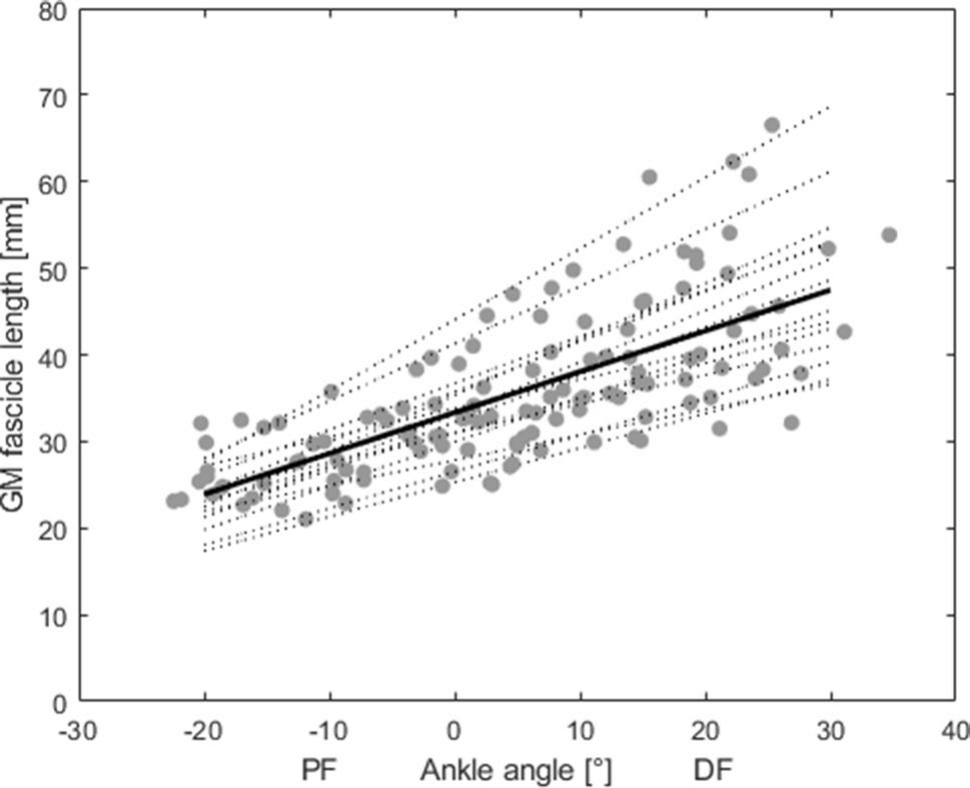
Mean (solid black) and participant specific (grey) relationships between ankle joint angle and gastrocnemius medialis (GM) fascicle length. 0° ankle joint angle refers to neutral foot position; *DF* dorsiflexion, *PF* plantar flexion angles.

With increasing dorsiflexion, GM fascicle length during MVC increased linearly (Fig. 4). Individual R^2^ for the linear fits used to describe the relationship between GM fascicle length and ankle joint angle ranged between 0.75 and 0.97 (on average 0.89 ± 0.06).

## Discussion

The purpose of this study was to investigate the influence of different literature based AT MA-a-functions on the estimation of TS force. We found significant changes in the magnitude of calculated forces, as well as significant differences regarding the shape of the resulting F-a-r.

In the present study, the experimentally assessed torques were in line with the literature^38,39^. We found a significant linear relationship between GM fascicle length and ankle joint angle which is in line with the literature^37,40^. Consequently, we can assume that the filament overlap contributes significantly to the force–length curve. Therefore, the influence of different AT MA-a-functions on the estimated F-a-r has direct implications on the estimated GM force–length relation.

Typically, for single-joint contractions, the TS was suggested to exclusively operate on the ascending limb of the F-a-r^40–42^. Yet, some participants of this study showed a clear descending limb towards more dorsiflexed positions (Fig. 1e–g) while others solely operated on the ascending limb (Fig. 1c,d). Previous studies reported similar or even more extreme variations in individual plantarflexion F-a-r or torque–angle relation in single-joint^41,43,44^ and multi-joint experimental setups^42^. This confirms that literature-based F-a-r characteristics should be used with caution for generalization.

There were large differences in the magnitude of calculated forces between different AT MA-a-functions used (Table 1, Fig. 3) with mean peak forces ranging from 4312 ± 1156 to 9947 ± 2194 N. Thereby, the geometric imaging method (2D) seemed to show most consistent and also largest AT MAs (Fig. 2a), resulting in the smallest calculated peak forces (on average 5993 ± 1176 N). In contrast, the tendon excursion method showed greater variability in AT MA magnitudes and curvature of the presented AT MA-a-functions (Fig. 2-b). This could be due to methodological differences, as the different working groups either disregarded^13^, accounted for^34^ or used an alternative method (i.e. measuring calcaneus bone excursion) to work around^37^ the impact of increasing passive TS forces on AT length in dorsiflexed ankle joint positions. The AT MA-a-functions based on the UsKin method were notably smaller than those obtained by the geometric imaging method (Fig. 2c), thus, resulting in higher forces. It seems that the determination of the AT MA-a functions, and therefore TS force estimation, is method-dependent^13^, especially for the tendon excursion method.

However, some of differences in AT MA magnitude could also be explained by the individual differences of the participants, as AT MA can be affected by body size^8,33^, contraction intensity^12,45^, the general morphology/anatomical position of the bones^35^ or gender^46^. The latter could especially be problematic when analyzing female participants^33^, as most studies used male participants (Table 1). Concerning a dependence of AT MA on the contraction intensity, some literature reports increased AT MAs during MVCs compared to rest^12,47^. Although there’s also contradicting work showing no effect of contraction intensity^36,37^, there is good reason to assume that AT MA changes (at least slightly) with varying contraction intensity. E.g. the thickening of the muscles during contraction likely shifts the AT further away from the joint rotational axis^12,47^. Using AT MA-a-functions determined during rest therefore increases the risk of overestimated muscle forces. We recommend to use participant specific AT MA-a functions in order to reduce the risk of misinterpretation of in *vivo* forces due to interindividual differences.

Especially, when analyzing TS force during movement with varying contraction intensities and ankle joint angles, like human locomotion^48,49^, the AT MA-a-function should be determined by a methodology sensitive to joint rotation and contraction intensity. The tendon excursion and geometric imaging methods do not seem to be feasible for this task. However, it should be mentioned that variations of both methods may allow for some accounting of these issues. Yet, the UsKin method as proposed by Manal et al.^16^, or variations of it^17^, might be the best solution. Still, the UsKin method has some limitations. Marker placement and the impact of skin movement artefacts are critical, as the rotational axis of the ankle joint is defined by markers placed on the medial and lateral malleolus. Further, shifts in center of rotation of the ankle joint cannot be detected. Moreover, this approach assumes that the ankle joint axis runs from medial to lateral malleolus. However, the rotation axis of the ankle joint with its six degrees of freedom is not in a fixed location but changes constantly^50^. Therefore, Wade et al.^20,21^ used an instantaneous axis of rotation derived from relative motions of the foot and shank segment to calculate the AT MA. Their results indicate that using a functional axis for ultrasound-based geometric estimates of AT MA shows a greater variation with changes in either joint angle or loading condition when compared to using anatomical landmarks (malleoli) to define the ankle’s rotation axis^20^. Still, using a fixed axis of ankle rotation might be preferable during dynamic movement, as it is applicable over a greater range of ankle motion^21^. Yet, despite those limitations, the UsKin method seems to be most promising for future in vivo studies as it is not limited by range of motion, accounts for inter-individual AT MA differences, and is sensitive to influences of joint rotation and varying contraction intensities. Rightfully so, this ultrasound-based geometric method has emerged to a common approach to determine the dynamic AT MA within recent years^17,20–25,27^.

Realistic calculation of TS forces is not only a prerequisite to determine physiological force–length data but also to determine realistic muscle properties including force–velocity relations and elastic properties of muscles from measured external reaction forces or joint torques^51–53^. Therefore, it is essential to evaluate which AT MA-a-functions lead to realistic TS force values. Only few studies have directly measured AT forces to allow such a validation. Direct AT force measurements during running showed peak forces at roughly 9000 N^2^. All but one AT MA-a-function used in this study led to notably lower estimated peak forces (Table 1). However, this is not necessarily a systematic underestimation of force, as the tasks at which the forces were assed in this study were isometric fixed-end single-joint MVCs versus dynamic multi-joint leg-extensions when AT forces were measured directly^2^. From previous work it is known that plantarflexion torque capacity during multi-joint leg-extensions, like in running, is increased compared to isolated single-joint plantarflexions^42^. Direct measurements of AT force during fixed-end single-joint MVCs are reported by Arndt et al.^1^, using an optic fiber pierced through the AT. In that study, AT forces of ~ 2900 N were recorded in neutral ankle joint position with the knee 30° flexed. This force is lower than what was found for the same ankle joint angle in the current study (between 3081 ± 402 and 5253 ± 685 N). However, a direct comparison with the current study is not possible as a 30° knee flexion results in a reduced fascicle length of the bi-articular parts of the TS (i.e. gastrocnemius lateralis and medialis) which is less favorable regarding their force capacity due to the force–length relation. For fixed-end single-joint MVCs at neutral ankle joint position a 30° knee flexion was shown to lead to a reduced maximum TS force capacity of ~ 10–12%^54,55^. Accounting for this factor would put three of the 15 estimated TS peak forces within a reasonable range of the value obtained through direct measurement. However, since Arndt et al.^1^ only measured three subjects this is highly speculative as net torque output is highly individual and therefore, is not comparable between studies with such a low number of participants. Alternatively, muscle force may be estimated from PSCA and estimates of muscle-specific tension^28^. However, both factors show large variation for TS in the published literature^7,56–60^. Further, the direct measurement approach^1,2,6^ as well as the muscle force estimation via PCSA and muscle-specific tension^28^ require an assumption of the AT MA in order to calibrate the buckle/optic fiber transducer or to calculate the muscle forces, respectively. Therefore, a statement of correctness of the used MA-a-functions based on prediction of “real” muscle forces cannot be made at this point.

Recently, shear wave speed measurements have been used to assess AT stress during plantarflexion contractions^24,61^. In order to estimate tendon force solely on shear wave speed, this newly developed technique requires the knowledge of effective tissue density, which cannot be measured in intact tendons^24^. Hence, a subject-specific measurement is needed to calibrate shear wave speed with related tendon force. In addition to joint torque (either measured by dynamometry or estimated by inverse dynamics), this calibration requires an information about the AT MA for the specific ankle joint angle^24^. Thus, this technique still depends on an estimation of the AT MA. However, this estimation is only needed in a single joint configuration. Due to the linear relationship of shear wave speed and AT force^62^ shear wave speed can then be used to estimate AT force at any given ankle joint angle or contraction condition. This technique would therefore not require any assumptions regarding the AT MA-a-function or F-a-r in order to estimate the force–length relationship of the TS, thus removing two major sources of uncertainty. Based on a methodologically sound calibration including subject-specific moment arms, this is a promising approach to study in vivo muscle–tendon unit force during simple as well as complex movements.

Two of the AT MA-a-functions^10,35^ showed a more rapid decline towards the more dorsiflexed positions (Fig. 2d). For both, this resulted in a purely ascending F-a-r without a plateau within the tested range of motion (Fig. 3a,e). All other MA-a-functions resulted in F-a-r having a plateau region. ANOVA statistics identified a significant interaction between the factors ankle position and MA-a-function Accordingly, the point at which the plateau phase of the F-a-r started was shifted by up to 10° depending on the MA-a-function used. Our results showed a linear relation between fascicle length and ankle joint angle (Fig. 4). Thus, AT MA related shifts of the plateau region observed in the F-a-r inevitably also occur in the force–length relation. In locomotor performance, where muscle function close to/within the plateau region of the force–length relation is regarded as favorable^48,49^, the uncertainty about its exact beginning likely has significant implications for the interpretation of task related muscle function such as movement efficiency. This underlines the risks of using literature based AT MA-a-functions for generalization without caution as not only magnitude of estimated forces, but also—maybe even more important—the shape of the F-a-r and force–length relation is clearly affected by this.

## Limitations

It should be noted that most studies considered in the current study focused on AT MAs around the neutral ankle joint position with a tendency to prioritize plantarflexed positions (Table 2). Therefore, AT MA in dorsiflexed positions had to be estimated by means of extrapolation, which is prone to error, especially with AT MA measured in only few ankle joint angles (Table 2). Additionally, the co-contraction by the main antagonist (tibialis anterior) was controlled via EMG but not taken into account for TS force calculation. However, Raiteri et al.^63^ showed a negligible effect of co-contractions during fixed-end plantarflexions. Further, this unaccounted factor for the determination of the individual torque–angle relation does not influence the main findings of this study (i.e. significant differences in shape and magnitude of estimated TS forces after applying the different AT-MA-a functions).

In this study, it was assumed that the TS constantly contributes 77% of the total plantarflexion moment. However, this might be an over simplistic model, as the contribution of the six additional ankle plantarflexors that are not attached onto the AT (plantaris, tibialis posterior, flexor halluces, longus, flexor digitorum longus, peroneus brevis and peroneus longus) have independent tendons, each with their individual MA-a-function.

The force-angle relations determined in this study rely on the assumption that muscle activity was constant across all joint angles. However, it might be argued that the decreasing forces in the more plantarflexed ankle joint positions are partly due to the participant’s inability to perform MVCs in this uncomfortable close to end-range position^64^. In order to prevent this, all participants attended a familiarization session to get accustomed to the setup. Further, only the highest active torque value out of two trials was considered for the analysis. This protocol was rewarded as our EMG data did not show any significant differences in muscle activity throughout the entire range of motion, except for soleus muscle at the most plantarflexed position. We believe the reduced soleus EMG level in this position was due to limited signal detection rather than reduced voluntary muscle activity. During movement, the surface electrodes change their relative position to the muscle belly due to the muscles sliding underneath the skin. For the gastrocnemius lateralis and tibialis anterior, both presenting a large muscle belly accessible through surface EMG, this only seems to have marginal effects on the observed muscle activity, as no matter the ankle joint position, the electrode constantly stays close to the midpoint of the muscle belly. In contrast to that, the electrode location on the soleus muscle (placed according to the SENIAM guidelines) must be relatively close to its distal tendinous part as its majority is covered by the gastrocnemii. Thus, especially in a plantarflexed position surface electrodes hardly covered the muscle belly but possibly moved over the tendinous part of the soleus muscle, explaining the reduced EMG levels.

## Conclusion

This study shows that TS force estimations are strongly affected by the choice of literature-based AT MA-a-function. This concerns the estimation of maximum muscle force, the corresponding joint angle for optimum force capacity, and the working range of the TS on the F-a-r. At this point we cannot identify the most accurate AT MA-a-function and thorough validation using direct force measurements is required. Regardless, for researchers interested in estimation of TS forces, the use of individualized AT MA measurements based on a hybrid ultrasound and 3D-kinematics approach may be recommended. This method accounts for inter-individual AT MA differences, identifies influences of joint rotation and can be easily used with varying contraction intensities.

## Data Availability

The datasets generated during and/or analyzed during the current study are available from the corresponding author on reasonable request.
